# The effects of talus control foot orthosis in children with flexible pes planus

**DOI:** 10.1186/1757-1146-7-S1-A119

**Published:** 2014-04-08

**Authors:** Bong-Ok Kim, Soo-Kyung Bok, So-Young Ahn

**Affiliations:** 1 Department of Rehabilitation Medicine, School of Medicine, Chungnam National University

## Objective

To identify the therapeutic effect of the talus control foot orthosis(TCFO) in children with severe flexible flat foot.

## Material and methods

TCFO is foot orthosis which combines inverted rigid foot orthosis(RFO) with broad upright portion that covers and protects the talonavicular joint, rising well above the navicular.

Fourty children were participated in this study who had been diagnosed as flexible pes planus, and had more than two consecutive radiologic studies. The anteroposterior talocalcaneal angles (APTCA) and lateral talocalcaneal angles (LTTCA), the lateral talometatarsal angles (LTTMA) and the calcaneal pitch (CP) of both feet were measured to evaluate foot alignment. Severe flexible pes planus was diagnosed when Beighton hypermobility score was greater than 4 points and when either of the feet had greater than 10 degrees valgus of resting calcaneal stance position angle and indicators showed greater than 55 degrees in APTCA and LTTCA, greater than 10 degrees in LTTMA, lesser than 10 degrees of CP.

Twenty children with flexible flat foot were fitted with a pair of RFO and another 20 children were fitted with TCFO. They were recommended to put on orthosis more than 8 hrs a day, to walk with heel strike at initial contact and reciprocal arm swing to normalize gait pattern. The follow up clinical evaluation with radiologic study was done after 12 months.

## Result

With TCFO, all radiologic indicators changed toward corrective direction than with RFO. There were statistically significant improvements in CP and RCSP in both groups. (p < 0.05) In TCFO group, APTCA, RCSP improved significantly compared with RFO group.

## Conclusion

This study showed that TCFO is effective in the treatment of children with severe flexible pes planus than RFO. The evaluation with long term follow up of radiographic study would be necessary to confirm the therapeutic effect of TCFO in children with pes planus.

